# The accuracy of robotic-assisted zygomatic implant placement: a systematic review and meta-analysis

**DOI:** 10.1186/s12903-026-07816-7

**Published:** 2026-04-10

**Authors:** Shengchi Fan, Thidarat Singkhorn, Gustavo Sáenz-Ravello, Nikos Mattheos, Rubén Davó, James Chow, Bilal Al-Nawas

**Affiliations:** 1 Department of Oral and Maxillofacial Surgery, Plastic Operations, University Medical Center Mainz, Mainz, Germany; 2 Oral Surgery and Implantology, Faculty of Medicine and Health Sciences, University of Barcelona, Barcelona, Spain; 3 Oral and Maxillofacial Surgery and Digital Implant Surgery Research Unit, Faculty of Dentistry, Chulalongkorn University, Bangkok, Thailand; 4 Faculty of Dentistry, Center for Epidemiology and Surveillance of Oral Diseases (CESOD), Universidad de Chile, Santiago, Chile; 5 Department of Dental Medicine, Karolinska Institute, Stockholm, Sweden; 6 Department of Implantology and Maxillofacial Surgery, Vithas Davo Instituto Dental, Hospital Medimar Internacional, Alicante, Spain; 7 Dental Implant Surgery Centre, Hong Kong, China

**Keywords:** Zygomatic implant, Computer-assisted implant surgery, Robotic surgery, Guided surgery, Navigation

## Abstract

**Background:**

This is the first systematic review and meta-analysis aimed at assessing the current status of robotic-assisted implant surgery (r-CAIS) in zygomatic implant (ZI) placement, with a particular focus on deviations between planned and placed implant positions.

**Materials and methods:**

The review protocol was specified and registered in PROSPERO (CRD42024616949). All primary studies were considered eligible, including in vitro studies, clinical studies, and hybrid experimental–clinical studies. Electronic and manual literature searches were conducted through April 2025 in PubMed/MEDLINE, Scopus, the Cochrane Library, and Web of Science. The primary outcome was planned/placed deviation with or without other implant placement techniques.

**Results:**

Ten studies (seven in vitro, two clinical, and one hybrid experimental–clinical) involving 185 ZIs in 83 models /patients were included. Of these, nine studies were eligible for quantitative synthesis, contributing 161 ZIs placed using r-CAIS and 97 using dynamic CAIS (d-CAIS) to the pooled mean meta-analysis. The pooled mean ZI deviations from the r-CAIS group were 1.04 mm (95% CI: 0.74–1.35 mm) at the entry point and 1.47 mm (95% CI: 1.07–1.87 mm) at the exit point, and angular deviations were 1.22° (95% CI: 0.82–1.62°). The pooled mean ZI deviations from the d-CAIS group were 1.30 mm (95% CI: 1.00–1.60 mm) at the entry point and 2.02 mm (95% CI: 1.81–2.23 mm) at the exit point, and angular deviations were 2.00° (95% CI: 1.33–2.67°). Significant differences in deviation were observed at the exit and angular levels in favor of r-CAIS.

**Conclusions:**

r-CAIS demonstrates significantly superior accuracy compared with d-CAIS in ZIs placement. However, as r-CAIS remains at an early stage of clinical integration and only limited clinical studies have been reported, its long-term applicability requires further validation through cadaveric and well-designed prospective clinical investigations.

**Supplementary Information:**

The online version contains supplementary material available at https://doi.org/10.1186/s12903-026-07816-7.

## Introduction

 P-I Brånemark revolutionized dental implantology by introducing the concept of the zygomatic implant (ZI), utilizing the zygomatic bone for implant anchorage to address the challenges associated with maxillary defects and severe atrophic edentulism [1]. The use of ZIs has demonstrated high survival rates, with numerous studies reporting consistent success over follow-up periods exceeding 10 years [2, 3]. As ZI protocols have evolved, their primary advantage has shifted significantly—from merely eliminating the need for extensive bone grafting to enabling immediate prosthetic rehabilitation on the day of surgery [4]. The approach focuses not only on restoring oral function but also on significantly enhancing patients’ quality of life [3].

Nowadays, digital workflows are integrated into nearly every step of implant therapy [5–7]. In ZI treatment, computer-assisted implant surgery (CAIS) has streamlined surgical planning and improved the precision of implant positioning [8]. Real-time navigation systems further enhance this process by providing continuous visualization of the implant trajectory [9]. A systematic review demonstrated that the use of CAIS systems in ZI surgery yields clinically acceptable outcomes in terms of average deviations [10]. However, despite the favorable mean deviation, substantial outliers were also reported. Surgeons should remain alert to potential deviations and complications, regardless of the chosen mode of guidance.

Given the extra-long trajectory of ZIs, even small deviation while drilling at the entry point can lead to substantial deviation at the apex within the zygomatic bone [11]. To address the limitations of manual operations, robotic-assisted implant surgery (r-CAIS) in ZI approach is emerging, offering the potential to achieve a high degree of precision [12]. In 2018, the first ZI robotic prototype, developed by Fan et al., [13] demonstrated the feasibility of using a robotic system to place one ZI on each model’s bilateral zygoma. Subsequently, the same group has continued to improve and refine the system to enhance its accuracy and practicality to perform a quad zygoma approach [14]. In 2023, clinical case reports employed different robotic systems to assist in ZI placement, achieving clinical advancement [15, 16].

With the rapid progression of technological innovation, r-CAIS appears to offer greater accuracy in dental implant than other modes of guided CAIS [17]. However, it remains unclear whether robotic system-assisted ZI placement has progressed beyond a conceptual framework toward a clinically feasible modality. Therefore, the present systematic review aims to assess the status of r-CAIS in ZI surgery, with a particular focus on deviations between planned and placed implant position.

## Methods

### Prospero registration

This systematic review was conducted according to the Cochrane Collaboration guidelines and followed the Preferred Reporting Items for Systematic Reviews and Meta-Analyses (PRISMA) guidelines [18]. The review protocol was specified and registered in PROSPERO (International Prospective Register of Systematic Reviews) under registration number CRD42024616949.

### PICO question

P: Patients with severely atrophic edentulous maxilla.

I: ZI placement using r-CAIS.

C: ZI placement using s-CAIS, d-CAIS, non-guided CAIS or non-comparison.

O: Three-dimensional deviations (entry, exit, and angular) between planned and placed ZI.

### Eligibility criteria

Articles that met the following criteria were included in this systematic review: (1) All primary studies, including clinical (i.e., randomized clinical trials (RCTs), prospective and retrospective cohort studies, case-control studies, case series, and technical note), “human”, “model”, and “cadaver” studies that reported the accuracy of r-CAIS of ZI; (2) studies reporting accuracy as the primary outcome; and (3) studies reporting the exact deviation between the pre-surgical planning and the final position of the ZI. (4) To comprehensively assess the current state of the field, case reports and technical notes were also included if they reported relevant accuracy metrics.

However, only studies with more than five subjects or at least ten ZIs placed were included in the quantitative meta-analysis to ensure adequate sample size and improve the statistical stability and reliability of deviation estimates.

Therefore, the exclusion criteria were: (1) studies only assessing virtual, augmented reality, or virtual placement, and (2) studies lacking measurable clinical outcomes.

### Information sources and search strategy

A comprehensive electronic and manual literature search was conducted until April 2025 by two independent reviewers (S.F and T.S).

Databases searched included PubMed/MEDLINE, Scopus, Cochrane Library, and Web of Science.

Boolean operators “AND” and “OR” were combined with the following terms/MeSH/keywords: (robot*[Title/Abstract]) AND (“zygomatic implant”[All Fields] OR “zygomatic implants”[All Fields] OR “zygoma implant”[All Fields] OR “zygoma implants”[All Fields] OR “zygomatic fixture”[All Fields]). Manual searches were performed in Google Scholar, references of selected articles, and journal issues published since 2018. Only English-language articles were included. Inter-rater agreement was evaluated using Cohen’s Kappa statistic in Microsoft Excel 2022 (Microsoft Corporation, Redmond, USA) and interpreted according to Landis and Koch criteria.

### Data extraction

Two independent reviewers (S.F and T.S) screened studies against the PICOs criteria and extracted data, which were subsequently verified by two peer reviewers (G.S and N.M).

Data extracted included: author(s), year of publication, study design, robotic system, number of patients/models and implants, indications (hybrid or quad zygoma) [19], surgical approaches, measured outcomes, conclusions, and mean ± SD of deviations. When necessary, corresponding authors were contacted for clarification of missing or unclear information.

### Risk of bias

Two independent reviewers (S.F and R.D) performed the risk of bias assessment.

For in vitro studies, the QUIN (Quality Assessment Tool for In Vitro Studies) was employed to evaluate the risk of bias across five domains [20]: sample preparation, measurement procedures, operator involvement, reporting quality, and data analysis.

For clinical studies, the ROBINS-I framework was used to assess risk of bias across seven domains, including bias due to confounding, participant selection, classification of interventions, deviations from intended interventions, missing data, outcome measurement, and selection of reported results [21]. Each domain was rated as “low,” “moderate,” “serious,” or “critical” risk of bias. Discrepancies between reviewers were resolved through discussion until consensus was reached.

### Statistical analysis

Qualitative (narrative) synthesis was performed for all included studies. Quantitative synthesis was conducted for continuous outcomes using the individual as the statistical unit of analysis.

Because multiple implants were frequently placed within the same patient or experimental model, clustering effects were expected. To account for this dependency, intra-class correlation coefficients (ICCs) were applied to adjust the effective sample size. ICC values of 0.3–0.4 were assumed as conservative estimates, consistent with previous methodological recommendations for clustered implant-level meta-analyses in dentistry [22, 23], in order to avoid underestimation of variance and overprecision of pooled estimates.

The primary outcomes included entry deviation (mm), exit deviation (mm), and angular deviation (degrees). Because both non-comparative and comparative studies were eligible, two complementary random-effects meta-analytic approaches were applied. A single-mean model estimated the overall accuracy of robotic-assisted implant placement, whereas a mean-difference (MD) model compared deviations between robotic-assisted surgery and other implant placement techniques. Random-effects models were fitted using restricted maximum likelihood estimation.

Confidence intervals were calculated using the Hartung–Knapp adjustment. Between-study heterogeneity was assessed using the chi-squared test (*p* < 0.05), the I² statistic, and τ² estimates, with τ and τ² confidence intervals derived using the Q-profile method. Publication bias was evaluated by visual inspection of funnel plots and, when applicable, Egger’s regression test (*p* < 0.05). All analyses were performed using the “meta” package in R (version 4.5.0).

## Results

### Paper selection process

**Fig. 1:**
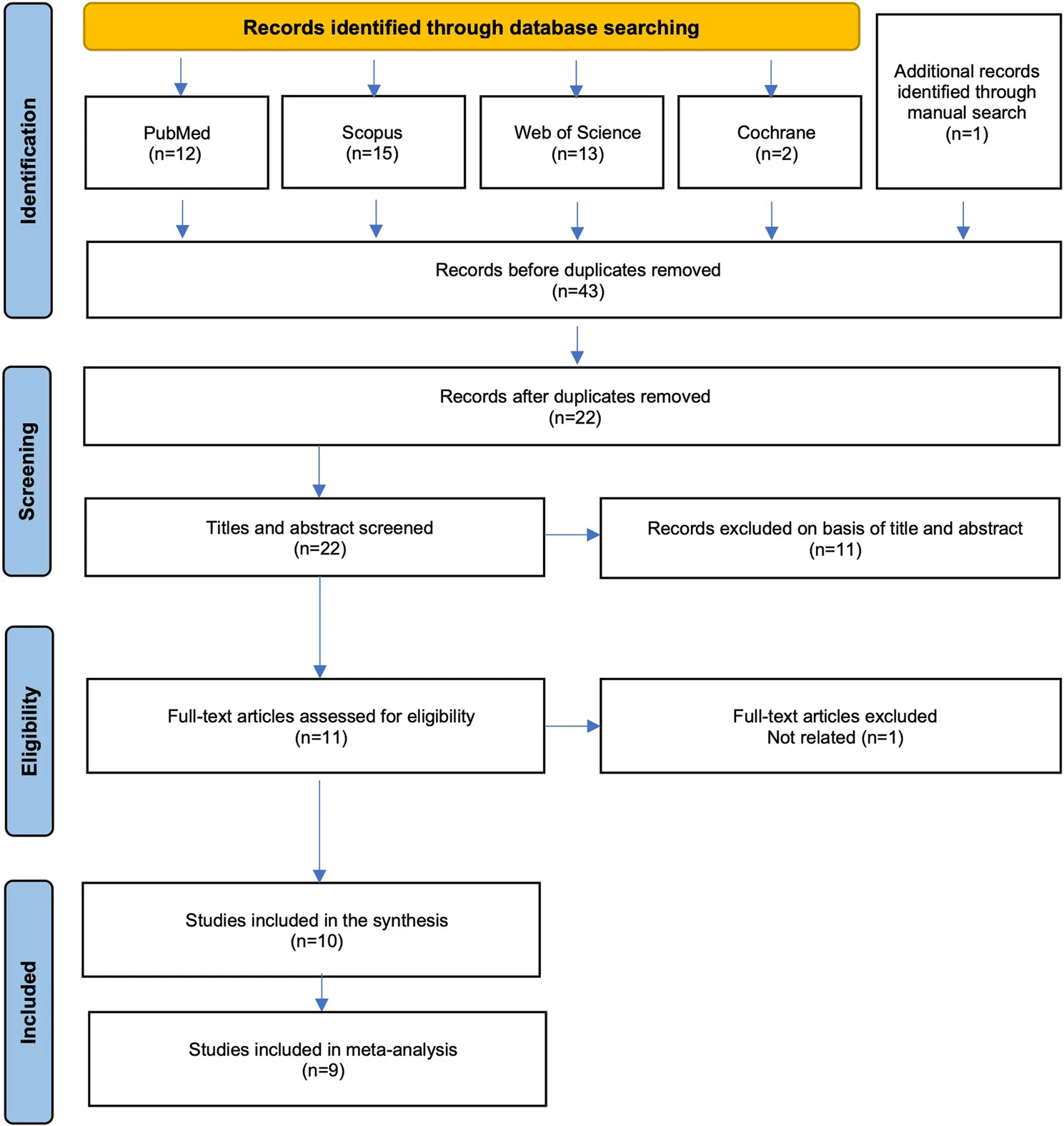
Flowchart of the study selection process

The last search of all databases was conducted in April 2025. 43 articles were retrieved through database searching, and one was retrieved through manual search. After removing duplicates (*n* = 21), 22 articles were screened using title-abstract-keyword reading, leaving 11 reports eligible (substantial agreement, κ = 0.9). After full-text reading and a subsequent search for relevant citations, 10 studies were included in this systematic review [13–16, 24–29] (100% of agreement among the reviewers) (Fig. 1). One clinical study was excluded due to not reporting r-CAIS deviation [30].

### Characteristics of the included studies

A total of ten studies, including seven in-vitro studies [13, 14, 22, 26–29], two clinical studies [16, 25] and one hybrid experimental–clinical study [15], evaluated robotic-assisted placement of ZIs, demonstrating varying levels of autonomy, accuracy, and feasibility. Across the ten included studies, a total of 83 models/patients received 185 ZIs, comprising both in vitro (75 models with 171 ZIs) and clinical (8 patients with 14 ZIs) investigations. Most ZIs were from Nobel Biocare (Nobel Biocare AB), with mean lengths ranging from 45 mm to 50 mm. The included studies originated from China (*n* = 8), France (*n* = 1), and the United Kingdom (*n* = 1). The main characteristics of the included studies are described in Table 1.

**Table 1:** The characteristics of the included studies

Author, years	Study type/ design	Number of model /Pt	Number of implant	Surgical approach	Indication	Comparison	Software	Implant	Mean ZI length(mm)	Robotic System	Robotic -arm	Registration	Conclusion
Fan, 2018 [13]	In vitro	6	12	NM	Classic	/	OralImplantPlanningV1.0, Shanghai, China	Nobel Biocare, Switzerland	50	Inhouse	UR3e, Universal Robots, Odense, Denmark	8 Fiducial	the automatic robotic system could achieve acceptable accuracy in ZIs placement.
Cao, 2020 [14]	In vitro	3	12	NM	Quad	d-CAIS (BrainLAB, AG, Germany)	CAPPOIS	Nobel Biocare, Switzerland	NM	Inhouse	UR3e, Universal Robots, Odense, Denmark	8 Fiducial	Pilot study confirmed the surgical robotic system’s accuracy and feasibility, outperforming manual surgery.
Li, 2023 [15]	In vitro	5	10	NM	Classic	/	NM	Nobel Biocare, Switzerland	NM	RemebotDent, Remebot, Beijing Rui Yi Bo Technology DICOM, Ltd.; China	UR3e, Universal Robots	Hard resin (Surgical Guide UV, HEYGEARS, Guangzhou, China)	The planning and procedures ensured accurate robotic ZI placement, unaffected by maxillary sinus lateral wall deviation
	Clinical	1	2										
Deng, 2023 [16]	clinical	6	8	Intra-sinus	Classic	/	Remebot; Beijing Parkway Wellcome Technology Co, Ltd	Nobel Biocare, Switzerland	46	Remebot; Beijing Parkway Wellcome Technology Co, Ltd	Remebot; Beijing Parkway Wellcome Technology Co, Ltd)	Custom surgical positioning piece	Task-autonomous robots offer high accuracy for ZI placement and eliminate human tremors
Deng, 2023 [24]	In vitro	10	26	AGA	NM	/	RemebotDent surgical design navigation software	Nobel Biocare, Switzerland	NM	Remebot; Beijing Baihui Weikang Technology Co., Beijing, China	UR3e, Universal Robots	8–12 ceramic beads were distributed separately as reference marks for registration.	Semi-autonomous two-stage robotic technique for ZIs is feasible and more accurate than the conventional one-stage method
Olivett, 2023 [25]	Clinical	1	4	Flapless	Quad	/	The ROSA one robot (Zimmer Biomet Robotics)	NM	NM	The ROSA one robot (Zimmer Biomet Robotics)	NM	Surface facial scan registration	Robot-assisted surgery offers precise, safe flapless ZI placement with reduced deviations
Al-Jarsha, 2024 [26]	In vitro	8	16 (8Ant./8Post.)	ZAGA	Quad	/	d-CAIS (NaviDent; ClaroNav Inc., Toronto, Canada)	Southern Implants, South Africa	NM	Inhouse	UR3e, Universal Robots	Six fiducials were placed in each plastic edentulous maxillary model (ZYG NM01 - D2 density; SelModels®	The errors of the dynamic navigation-guided robotic placement of zygomatic implants were within the clinically acceptable limits.
Fan, 2024 [27]	In vitro	5	20	NM	Quad	d-CAIS(NM) with AR glasses (HoloLens)	DentalHelper	Nobel Biocare, Switzerland	45	HoloLens 2 MR device	Hybrid robot (Zhejiang Zhihang Tianshu Medical Technology Co., Ltd., China)	Several mini-screws (φ1.7 mm × 10 mm) (Jianwei Co., Ltd, Shenzhen, China) were implanted into the alveolar bone	The hybrid robotic system with MR navigation achieved superior accuracy and stability vs. manual placement in phantom tests and shows promise for preclinical studies.
Guo, 2024 [28]	In vitro	28	55	NM	Classic	d-CAIS (Digital-Care Medical Technology Company Ltd., Suzhou, China).)	Mimics Research 21.0, Materialise, Belgium	Nobel Biocare, Switzerland	46.6	Remebot semiautonomous(Beijing Baihui Weikang Technology Co., Beijing, China)	UR3e, Universal Robots, Odense, Denmark	At least seven ceramic beads and a marker were installed on all models	The robotic system prevents ZI overextension but has slightly lower exit-side accuracy than dynamic navigation.
Chen, 2024 [29]	In vitro	5	10	Intra-sinus	Classic	d-CAIS (DCARER)	NM	Straumann AG, Basel, Switzerland	NM	(Remebot; Beijing Baihui Weikang Technology Co. Ltd.)	UR3e, Universal Robots, Odense, Denmark	A customized marker plate (Remebot; Beijing Baihui Weikang Technology Co. Ltd.) was designed and fixed to the palate with pins	For computer-guided ZI placement, task-autonomous r-CAIS was superior to d-CAIS in terms of accuracy

*AGA* Anatomically guided approach, *d-CAIS* Dynamic computer assisted implant surgery, *MR* Mixed reality, *NM* Not mentioned, *r-CAIS* Robotic computer assisted implant surgery, *ZI* Zygomatic implant

Two approaches (hybrid and quad) were used depending on the degrees of alveolar atrophy. The classic approach (placing one ZI on each side) was reported in 5 studies comprising 7 patient and 49 models with 107 ZIs. The quad approach (placing two ZIs on each side) was reported in 4 studies, including 1 patient and 16 models with 52 ZIs. In one of Deng’s [24] study, 26 ZIs placements in 10 models did not report the exact approach. Among the included studies, four directly compared r-CAIS with d-CAIS, while none reported comparisons with s-CAIS or freehand approaches. A total of 97 ZIs were placed across 41 models. The deviation values reported in each study are summarized in Table 2.

**Table 2:** The deviations reported in included study

Author, years	Number of ZI (subgroups)	Entry Point mm (Range)	Exit deviation mm (Range)	Angular deviation ° (Range)	Accuracy Analysis Tool (software)
Robotic computer-aided implant surgery					
Fan, 2018 [13]	12	2.34 ± 0.79 (1.25 −3.25)	2.57 ± 1.73 (0.21–4.23.21.23)	2.76 ± 1.39 (1.10 −5.01)	CBCT (NP)
Cao, 2020 [14]	12	0.79 ± 0.19 (0.53–1.08.53.08)	1.49±0.48 (0.75–2.50.75.50)	1.52 ± 0.58 (0.75–2.19.75.19)	CBCT (NP)
Deng, 2023 [16]	8	0.97 ± 0.50 (0.21–1.51.21.51)	1.27 ± 0.67 (0.35–2.13.35.13)	1.48 ± 0.61 (0.49–2.29.49.29)	CBCT (Remebot; Beijing Parkway Wellcome Technology Co, Ltd)
Li, 2023 [15]	10 (Model)	0.78 ± 0.34	0.80 ± 0.25	1.33 ± 0.41	CBCT (RemebotDent, Remebot, Beijing Rui Yi Bo Technology DICOM, Ltd.; China)
	2 (Patient)	0.83 (0.68–0.98.68.98)	1.10 (1.02–1.17.02.17)	1.46 (1.21–1.70.21.70)	
Olivetto, 2023 [25]	4	1.84 ±1.60 (0.85–4.22.85.22)	2.19 ±1.76 (0.91–4.70.91.70)	/	CBCT (Rosana planning software (Zimmer Biomet Robotics)
Deng, 2023 [24]	13 (Two-stage technique)	0.57 ± 0.19	1.07 ± 0.48	0.91 ± 0.51	CBCT (NP)
	13 (One-stage technique)	1.22 ± 0.76	2.13 ± 0.83	1.58 ± 0.88	CBCT (NP)
Chen, 2024 [29]	10	1.29 ± 0.46 (0.19–1.05.19.05)	0.88 ± 0.28. (0.25 −1.18)	0.92 ± 0.40 (0.18 - 1.59)	CBCT (Remebot, Beijing Baihui Weikang Technology Co. Ltd., Yizhime; DCARER)
Al-Jarsha, 2024 [26]	8	1.80 ± 0.96	2.80 ± 0.95	1.74 ± 0.92	CBCT (EvaluNav software)
Fan, 2024 [27]	20	0.88 ± 0.44	1.20 ± 0.66	1.54 ± 1.06	CBCT (The 3D Slicer platform)
Guo, 2024 [28]	55	1.53 ± 0.92 (0.21 −4.39)	2.39 ± 1.24 (0.13 −4.90)	1.55 ± 0.82 (0.16 −3.17)	CBCT (The Dcarer® Implant Dynamic Navigation System)
Dynamic computer-aided implant surgery					
Cao, 2020 [14]	12	0.63 ± 1.25 (0.53–1.08.53.08)	2.26±0.32 (1.83–2.70.83.70)	2.07 ± 0.30 (1.65–2.48.65.48)	CBCT (NP)
Chen, 2024 [29]	10	1.29 ± 0.46 (0.85 - 1.98)	1.96 ± 0.46 (1.22 - 2.70)	2.03 ± 0.53 (1.01- 2.73)	CBCT (Remebot, Beijing Baihui Weikang Technology Co. Ltd., Yizhime; DCARER)
Fan, 2024 [27]	20	1.45 ± 0.62	1.83 ± 1.00	2.48 ± 1.25	CBCT (3D Slice)
Guo, 2024 [28]	55	1.58 ± 1.25 (0.22 - 5.19)	1.83±1.25 (0.19 - 6.21)	1.50 ± 0.83 (0.08 - 4.01)	CBCT (Dcarer Implant Dynamic Navigation System)

*NP* Not reported

### Pooled mean Meta-analysis of Entry, Exit, and angular deviations

#### Subgroup Meta-Analysis of trueness (Mean Deviation) in r-CAIS and d-CAIS

**Fig. 2:**
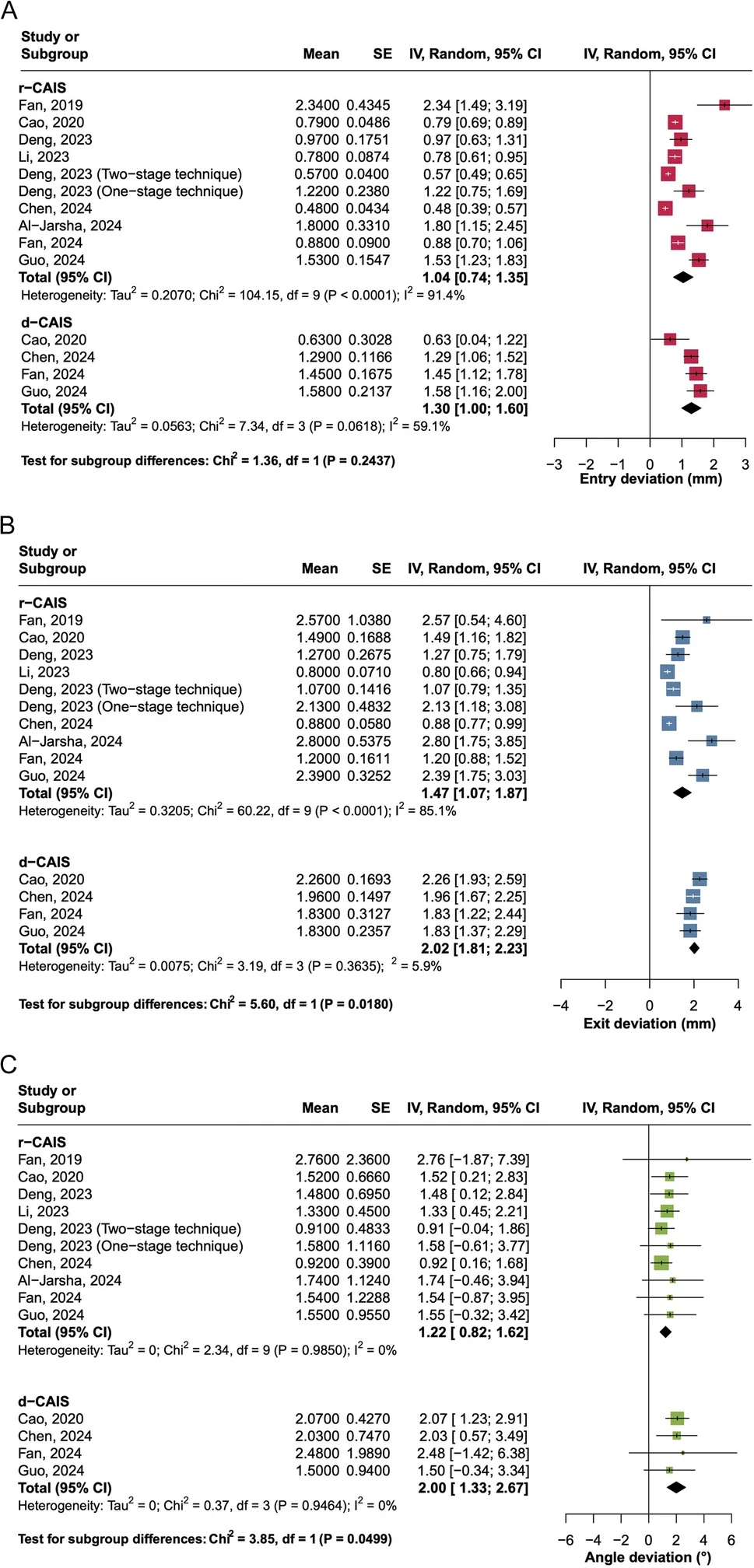
Forest plot representing the pooled mean deviation grouped by r-CAIS and d-CAIS in (**A**) entry deviation (**B**) exit deviation (**C**) angular deviation

A total of 9 studies, comprising 161 ZIs placed using r-CAIS and 97 using d-CAIS, were included in the pooled-mean meta-analysis (Fig. 2a-c). In the study by Li et al. [15], only the results from the model experiments were included, as clinical data were excluded due to limit to a single patient. Same excluded reason for the Olivetto et al. [25] that only one case was reported.

The pooled mean entry deviation was 1.04 mm (95% CI: 0.74–1.35 mm) for r-CAIS and 1.30 mm (95% CI: 1.00–1.60 mm) for d-CAIS, with no statistically significant subgroup difference (Chi² = 1.36, *P* = 0.2437).

For exit deviation, the pooled mean was 1.47 mm (95% CI: 1.07–1.87 mm) for r-CAIS and 2.02 mm (95% CI: 1.81–2.23 mm) for d-CAIS, demonstrating a statistically significant difference (Chi² = 5.60, *P* = 0.0180).

Regarding angular deviation, the pooled mean was 1.22° (95% CI: 0.82–1.62°) for r-CAIS and 2.00° (95% CI: 1.33–2.67°) for d-CAIS, also showing a statistically significant subgroup difference (Chi² = 3.85, *P* = 0.0499).

#### Comparative Meta-Analysis of trueness (Mean Deviation) between r-CAIS and d-CAIS

**Fig. 3:**
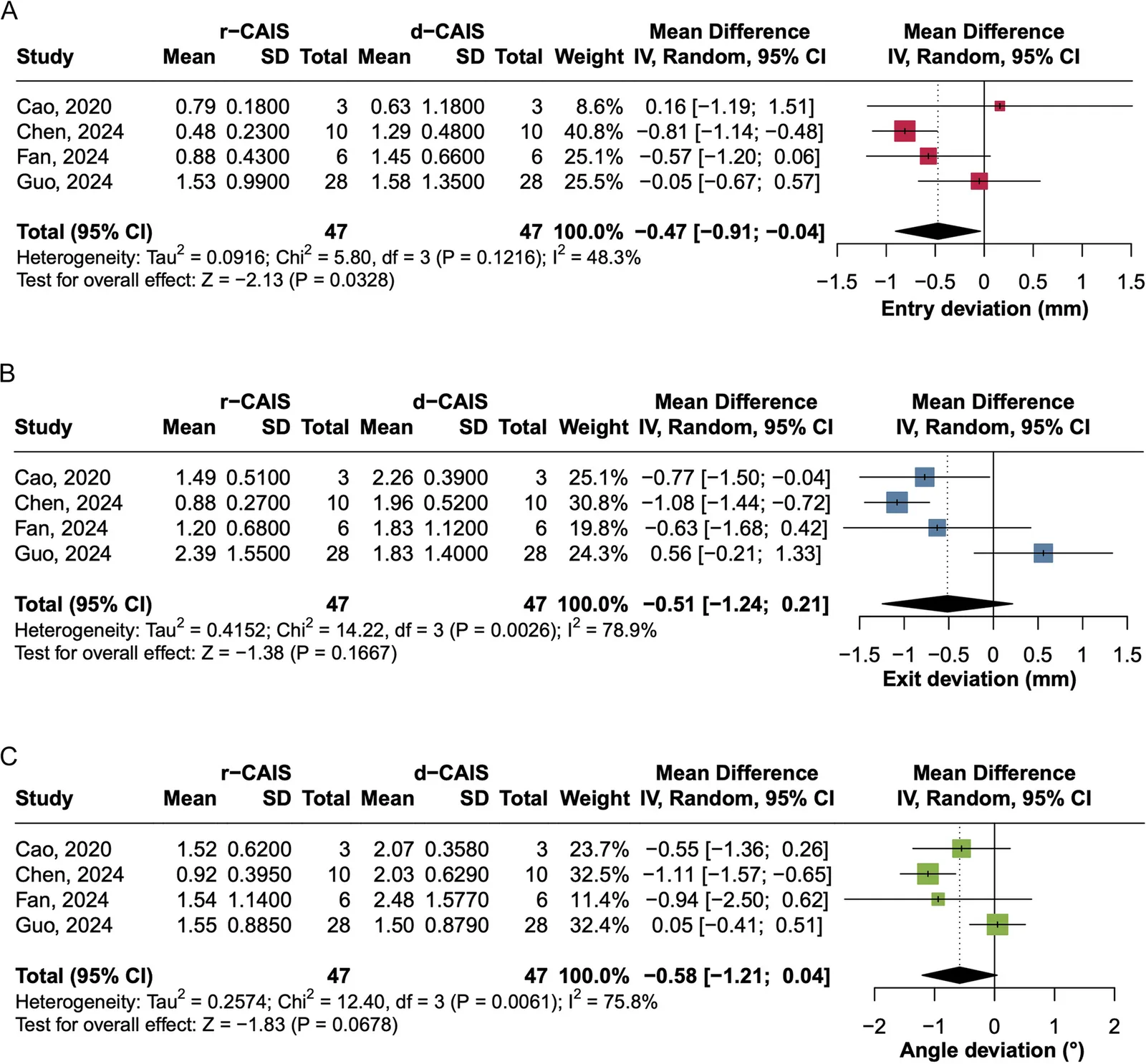
Forest plot representing the pooled mean deviation with comparative studies grouped by r-CAIS and d-CAIS in (**A**) entry deviation (**B**) exit deviation (**C**) angular deviation

A total of 4 studies, comprising 161 ZIs placed using r-CAIS and 97 using d-CAIS, were included in the pooled-mean meta-analysis (Fig. 3a-c). In the comparative meta-analysis, the pooled mean difference in entry deviation between r-CAIS and d-CAIS was − 0.47 mm (95% CI: −0.91 to 0.04 mm), showing statistically significant difference (*P* = 0.0328).

However, the exit deviation revealed a mean difference of − 0.51 mm (95% CI: −1.24 to 0.21 mm), which was not statistically significant (*P* = 0.1667). For angular deviation, the mean difference was − 0.58° (95% CI: −1.21 to 0.04°), indicating no significant difference (*P* = 0.0678).

### Assessment of publication bias

**Fig. 4:**
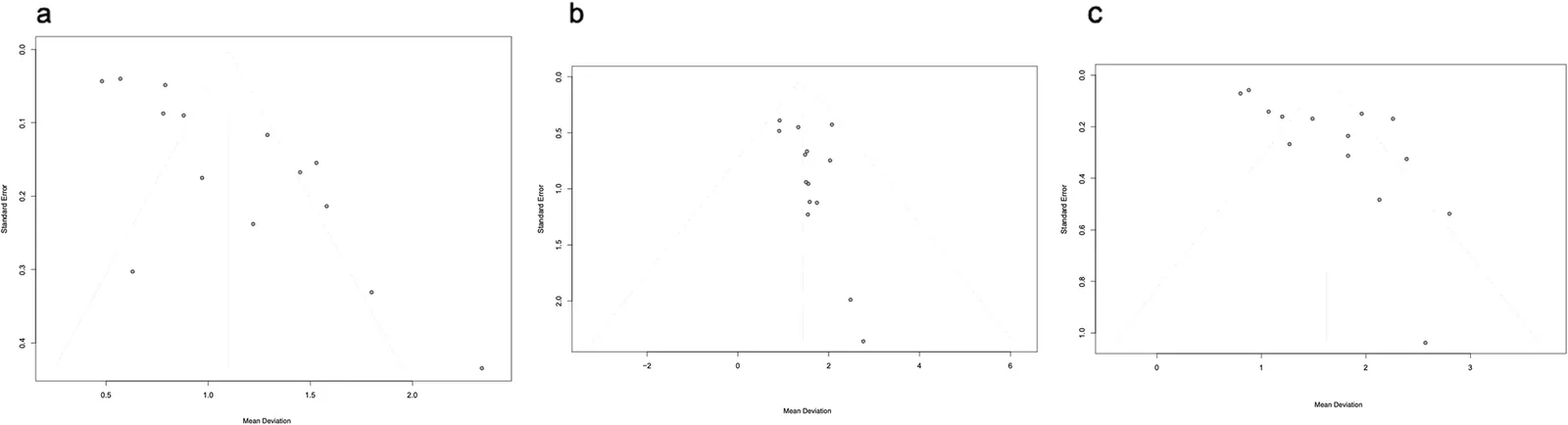
The funnel plot was used for the visual inspection of asymmetry in the r-CAIS for (**a**) entry, (**b**) exit, and (**c**) angular deviation with non-comparative studies

**Fig. 5:**
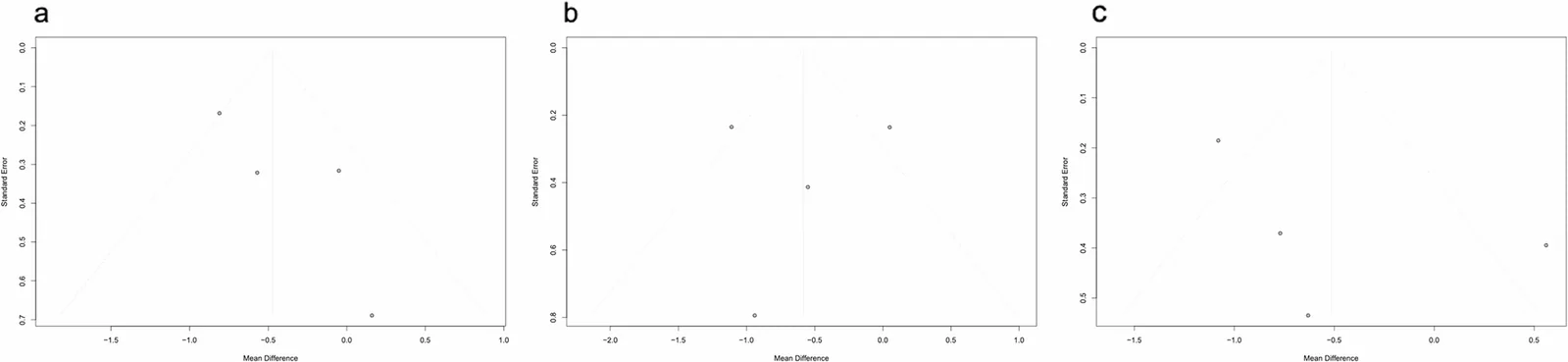
The funnel plot was used for the visual inspection of asymmetry deviation difference between r-CAIS and d-CAIS for (**a**) entry, (**b**) exit, and (**c**) angular deviation in comparative studies

The visual inspection of funnel plot asymmetry shows a tendency for asymmetry for both groups (non-comparison and comparison) for entry, exit and angular deviations (Figs 4 and 5).

### Risk of bias assessment

In-vitro studies demonstrated generally low risk of bias across sample preparation, measurement, operator, reporting, and analysis domains, with minor concerns in certain studies regarding measurement calibration and operator consistency (Table 3). Regarding clinical study, Deng et al. [16] demonstrated overall moderate risk of bias. No study was classified as high risk overall, supporting the reliability of the pooled findings (Table 4).

**Table 3:** Results of risk of bias of in-vitro studies by using the QUIN

Author	Sample Preparation	Measurement Procedures	Operator Involvement	Reporting Quality	Data Analysis
Fan et al. [13]	Moderate	Low	Low–Moderate	Moderate	Low–Moderate
Cao et al. [14]	Moderate	Low	Moderate	Moderate–High	Moderate
Li et al. [15]	Moderate	Low	Moderate	Moderate–High	Moderate
Deng et al. [24]	Moderate	Low	Low–Moderate	Moderate	Moderate
Fan et al. [27]	Moderate	Low	Moderate–High	Moderate	Moderate
Al-Jarsha et al. [26]	Moderate	Low	Moderate–High	Moderate	Moderate
Guo et al. [28]	Moderate	Low	Moderate–High	Moderate	Moderate–High
Chen et al. [29]	Low–Moderate	Low	Moderate	Moderate	Low–Moderate

**Table 4:** Results of risk of bias in clinical study by using ROBINS-I

ROBINS-I Risk of Bias Assessment for Deng et al. [16]							
confounding	selection of participants	classification of interventions	deviations from intended interventions	missing data	measurement of outcomes	selection of the reported result	Overall Risk of Bias
Moderate	Low	Low	Low	Low	Moderate	Moderate	Moderate

## Discussion

Taken together, the available evidence suggests that r-CAIS for ZI placement has progressed beyond a purely conceptual stage toward an emerging and potentially clinically feasible technique, demonstrating significantly greater accuracy than d-CAIS. When interpreted alongside previously published investigations on non-guided, static, and dynamic approaches, the pooled deviations observed with r-CAIS appear comparable or lower than those reported for conventional guidance methods [31, 32]. These findings may be explained by improved mechanical stability and trajectory control during the extra-long drilling pathway of ZIs, which could reduce cumulative deviation and enhance surgical predictability. Nevertheless, r-CAIS remains at an early stage of clinical integration, and its feasibility requires further validation through well-designed prospective clinical studies.

Although r-CAIS systems offer enhanced accuracy, it is critically dependent on system calibration, registration fidelity, and intra-operative stability—factors that are also essential in the performance of d-CAIS systems [29]. Among these, registration accuracy is the most critical determinant of overall performance. In edentulous arches, point-to-point registration is regarded as the gold standard [33, 34]. To achieve an acceptable registration error in navigation-guided surgery, the placement of 5–8 fiducial screws, widely distributed across the maxilla, has been investigated and recommended [35, 36]. In order to minimize the invasiveness of registration, instead of multiple bone screws insertion, 4 of the included in-vitro studies [15, 24, 28, 29] employed a screw-fixed plate combined with 7–12 of ceramic/pin fiducial for the registration method. However, the design and dimensions of the fixed plate in some studies appear to be impractical for clinical application due to its relatively large size. Furthermore, the fit and stability of the mounted plate are highly influenced by the degree of alveolar atrophy and the quality of the CBCT/intra-oral scan. From a clinical perspective, these factors may increase surgical setup time, complicate intra-operative workflow, and reduce usability in severely atrophic maxillae, thereby limiting the routine applicability of plate-based registration outside controlled experimental settings.

The surgical techniques for ZI placement have been well-documented in previous studies, which are generally classified into three approaches based on individual maxillary anatomy: intra-sinus, in the sinus wall, and extra-sinus [37–39]. While the relationship between ZI trajectories and the maxillary sinus has been extensively discussed in the context of postoperative sinusitis, it has been less frequently examined from a surgical and biomechanics perspective. ZI drilling instruments typically range from 60 to 100 mm in length, which increases the risk of tip deflection or bending when encountering resistance from the lateral cortical bone. This issue is more frequently encountered when the ZI is planned along an extra-sinus or sinus-wall trajectory. In such cases, an open flap approach is recommended when using the d-CAIS to provide certain surgical visibility. This allows verification of the drill’s axial alignment and ensures that the actual position of the drilling tip corresponds accurately with the real-time navigation display. Clinical study from Li [15] reported to use a custom-made of trigonometric drill for avoiding deflection while r-CAIS drilling sequence. In Deng’s in-vitro r-CAIS study [24], apical deviation in the intra-sinus group was significantly lower than in the other two approaches (*p* < 0.05), which might be biomechanically explained. For the modification of r-CAIS in ZI surgery, considerations such as drill length and robotic automatic force adjustment should be thoroughly investigated to reduce deviation and improve the potential for execution across all surgical approaches.

Most of r-CAIS utilizes either semi-active (Level 1) or task-autonomous (Level 2) systems, with level 1 offering guided surgeon control and level 2 enabling autonomous execution of predefined tasks under supervision [5]. In a recent systematic review, Pozzi et al. [17] reported that autonomous r-CAIS achieved highly accuracy, with deviations of 0.60 mm at the entry, 0.63 mm at the apex, and 1.24° in angulation—significantly more accurate than s- and d-CAIS. Additionally, the analysis included 27 full-arch rehabilitations involving 197 implant placements, providing supportive evidence for the clinical feasibility of robotic-assisted surgery in the rehabilitation of fully edentulous patients. For the current review, all the studies were used of level 2 robotic systems with clinical acceptable mean deviation. Regarding the range of errors, only seven studies [13–16, 25, 28, 29] reported the maximum deviation values, none of which demonstrated extreme outliers in any parameter compared to previous reviews on static and dynamic CAIS in ZIs placement. However, standardized reporting of accuracy metrics is strongly recommended in future publications to enable meaningful comparisons.

Beyond evaluating accuracy, the present systematic review also aimed to assess the current state of r-CAIS in ZI surgery. However, among the 10 included studies, only two were clinical studies with very limited subjects. Surprisingly, no cadaver study was conducted as a preliminary investigation on this topic. In contrast, both static and dynamic CAIS have been supported by preliminary cadaver studies to verify the feasibility of each technique in the early phase. Chrcanovic et al. [11] reported that conventional surgical template was not sufficient for achieving accurate ZI placement at the level of zygomatic bone, while Watzinger et al. [40] conducted the first cadaver study utilizing d-CAIS in ZI placement for defected maxilla, marking a significant step forward in the field. In the context of r-CAIS, sensory feedback during surgery differs substantially from surgeon-operated procedures, and the setup of the robotic arm is critical for achieving optimal autonomous performance. Factors such as mouth opening and mandibular dentition significantly affect the success of the drilling process. The selection between a straight or contra-angle handpiece, as well as the design of a customized handpiece holder and reference markers, must be carefully planned [41]. Therefore, validation through cadaver studies prior to initiating clinical trials may be advisable.

This systematic review has several limitations. First, the current evidence base remains dominated by in vitro studies, with only a limited number of clinical investigations available, which restricts the generalizability of the findings to routine clinical practice. Second, substantial heterogeneity existed among robotic platforms, registration protocols, and levels of system autonomy, potentially influencing accuracy outcomes and limiting direct comparability across studies. Moreover, most included studies focused primarily on technical accuracy metrics, while clinically relevant endpoints such as implant survival, complications, and patient-reported outcomes were rarely reported. From a practical perspective, the clinical adoption of r-CAIS may also be challenged by equipment costs, learning curve requirements, and increased workflow complexity. Finally, as this emerging technology is often evaluated in specialized centers with expert operators, learning-curve effects and performance bias cannot be excluded. These factors should be considered when interpreting the present findings.

## Conclusion

This is the first systematic review and meta-analysis to demonstrate that r-CAIS achieves significantly superior accuracy in ZI placement compared to d-CAIS. However, r-CAIS remains in a preliminary stage of clinical integration, and its feasibility warrants further validation through comprehensive, multidisciplinary evaluation.

## Supplementary Information


Supplementary Material 1.


## Data Availability

All data generated or analysed during this study are included in this published article and its supplementary information files.
