# Supplementary material for: The accuracy of robotic-assisted zygomatic implant placement: a systematic review and meta-analysis
Source: BMC Oral Health. 2026 Apr 10;26:778. doi: 10.1186/s12903-026-07816-7 (PMC13135268; doi:10.1186/s12903-026-07816-7)
Supplement: Supplementary file 1 — Supplementary Material 1. [file 12903_2026_7816_MOESM1_ESM.docx]

**Table S1.** Search queries

| **Database** | **Query** |
| --- | --- |
| PubMed | (robot*[Title/Abstract]) AND (“zygomatic implant”[All Fields] OR “zygomatic implants”[All Fields] OR “zygoma implant”[All Fields] OR “zygoma implants”[All Fields] OR “zygomatic fixture”[All Fields]) |
| Web of Science | ALL=(robot*) AND ALL=("zygomatic implant" OR "zygomatic implants" OR "zygoma implant" OR "zygoma implants" OR "zygomatic fixture") |
| Scopus | TITLE-ABS-KEY(robot*) AND TITLE-ABS-KEY("zygomatic implant" OR "zygomatic implants" OR "zygoma implant" OR "zygoma implants" OR "zygomatic fixture") |
| Cochrane Library | (“robot*”) AND ("zygomatic implant" OR "zygomatic implants" OR "zygoma implant" OR "zygoma implants" OR "zygomatic fixture") |
